# Lupus Enteritis: An Uncommon Presentation of Lupus Flare

**DOI:** 10.7759/cureus.18030

**Published:** 2021-09-16

**Authors:** Joanna Potera, Emmanuel Palomera Tejeda, Shilpa Arora, Augustine M Manadan

**Affiliations:** 1 Internal Medicine, John H. Stroger, Jr. Hospital of Cook County, Chicago, USA; 2 Rheumatology, Rush University Medical Center, Chicago, USA

**Keywords:** lupus enteritis, mesenteric vasculitis, systemic lupus erythematosus, lupus flare, gastrointestinal symptoms

## Abstract

Gastrointestinal (GI) symptoms are common in systemic lupus erythematosus (SLE) but are usually attributable to medication side effects, infections, or other underlying conditions. In rare cases, they are caused by the autoimmune process itself. In this report, we present two cases of lupus enteritis as the sole manifestation of lupus flare. We also provide a comprehensive review of available literature on this topic with a specific focus on clinical symptoms, complications, laboratory findings, histology, imaging findings, and therapies. Lupus enteritis is an uncommon manifestation of SLE. CT scan of the abdomen is the diagnostic modality of choice. The three major CT findings are target sign, comb sign, and increased mesenteric fat attenuation. Ascites is also commonly present. Corticosteroids and second-line immunosuppressants have been successfully employed in the treatment of lupus enteritis. Our cases highlight this unusual manifestation as the only symptom of active SLE. A high index of suspicion should be maintained when evaluating SLE patients presenting with GI symptoms to prevent diagnosis and treatment delays that could lead to serious complications such as bowel necrosis, perforation, and even death.

## Introduction

Systemic lupus erythematosus (SLE) is a multisystemic and chronic autoimmune disease. Its clinical course is usually fluctuating, characterized by intermittent flares involving virtually any organ or system of the body. Gastrointestinal (GI) symptoms are present in up to 40-50% of patients with SLE [1,2]. Nevertheless, GI involvement due to SLE activity is uncommon, often difficult to diagnose, and potentially life-threatening [1].

The term lupus enteritis refers to inflammation of the bowel wall due to SLE activity. Its pathogenesis may include immune complex deposition in the bowel wall or small vessel vasculitis [1]. It is reported in 0.2-5.8% of SLE patients and usually presents as nonspecific GI symptoms in the context of high disease activity with multiple organ involvement and systemic complaints [1-4]. It is very rarely seen as an isolated manifestation of the disease flare. Current knowledge of lupus enteritis is scarce and based mostly on case reports and case series. In this report, we discuss two cases and engage in a review of the available literature.

## Case presentation

Case 1

A 19-year-old female without any prior medical problems presented with four months of diarrhea, nausea, vomiting, epigastric pain, malaise, and weight loss. She had been initially evaluated at two different hospitals where she had been managed symptomatically with antispasmodic agents and proton pump inhibitors. Later on, she presented to our institution with no improvement in her symptoms.

**Figure 1:**
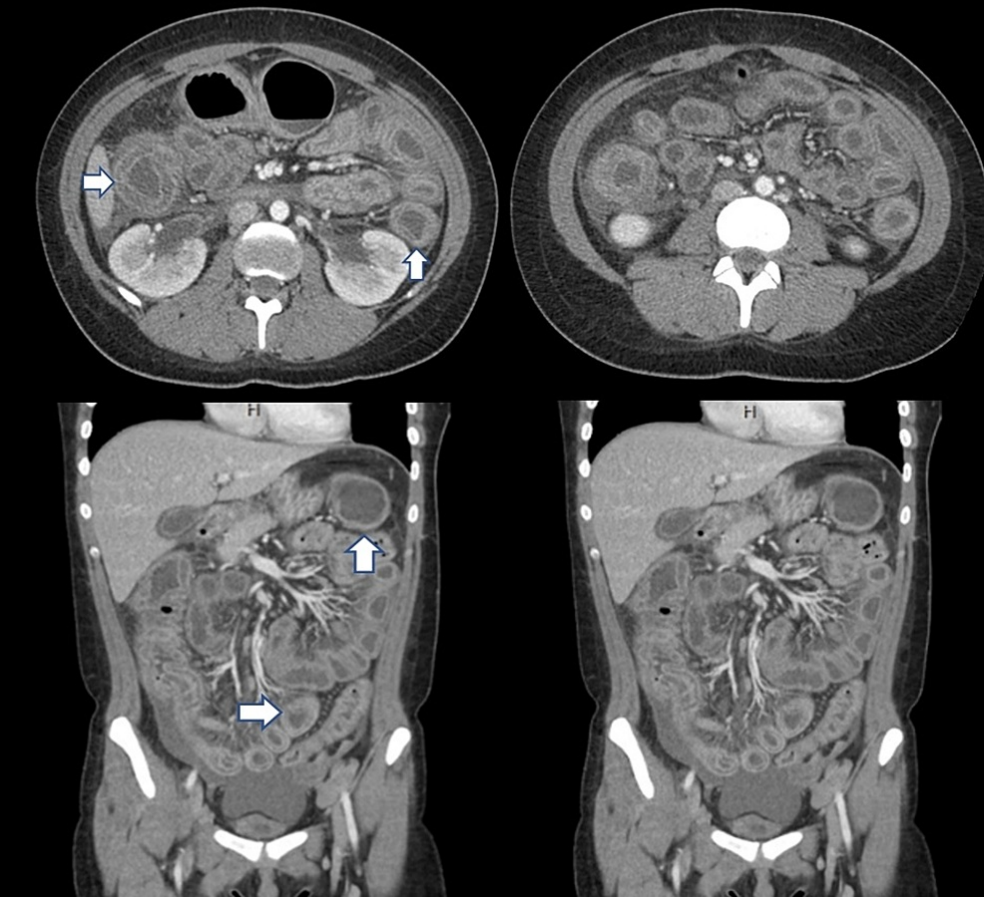
Contrast-enhanced abdominal CT scan - case 1 The scan revealed “target” or “double halo” sign (arrows) that represent circumferential thickening of the bowel wall secondary to submucosal edema. Both small and large intestines were affected in this case CT: computed tomography

Her family and social history were noncontributory. She was afebrile and normotensive, had no joint or skin symptoms, and on physical exam, her abdomen was mildly tender. Laboratory testing revealed hypomagnesemia (serum magnesium: 0.5 mg/dl), hypokalemia (potassium: 2.5 mEq/l), hypoalbuminemia (albumin: 3.4 g/dl), normocytic anemia (hemoglobin: 11.2 g/dl), lymphopenia (300 k/µL), and elevated C-reactive protein (CRP, 1.40 mg/dl). The infectious evaluation was negative (including Clostridioides difficile toxin, stool culture, and ova and parasites). A CT scan showed diffuse thickening and enhancement of the small and large bowel wall and a small amount of ascites (Figure 1). Colonoscopy and enteroscopy revealed nonspecific diffuse edema and erythema of the stomach and diffusely within the small bowel and colon, with biopsy showing mild chronic gastritis, nonspecific inflammation of the small intestine, and colon with focally increased intraepithelial lymphocytes. Despite symptomatic treatment, the patient did not show any improvement. Further evaluation revealed antinuclear antibodies (ANA) titer of >1:160, low complements (C3: <50 mg/dl and C4: 9 mg/dl), positive anti-double-stranded DNA (anti-dsDNA) of >300 IU/ml, SSA of >8, SSB of >8, anti-histone Ab of >5, and positive lupus anticoagulant. During the second week of hospitalization, the patient developed a faint malar rash and was diagnosed with SLE with the initial manifestation of lupus enteritis. Intravenous methylprednisolone was started with remarkable symptomatic improvement. She was transitioned to oral prednisone and tapered off over the next three months. Hydroxychloroquine and azathioprine were used as steroid-sparing agents. She remained asymptomatic at the six-month follow-up.

Case 2

A 49-year-old female was diagnosed with SLE in 2017 with manifestations of malar rash, photosensitivity, sub-nephrotic range proteinuria, anemia, lymphopenia, positive serologies, and hypocomplementemia. She was initially treated with prednisone and hydroxychloroquine with good response, but the patient later discontinued treatment and was lost to follow-up. Two years after the diagnosis, she was hospitalized with a one-month history of generalized abdominal pain and bloating, poor appetite, nausea, and vomiting. CT scan of the abdomen showed wall thickening of the duodenum and proximal jejunum with adjacent mesenteric edema and moderate ascites. Esophagogastroduodenoscopy revealed a non-obstructing Schatzki ring and no obvious mucosal changes to correlate with CT scan findings, as well as Barrett's esophagus without dysplasia. Colonoscopy was normal. Infectious evaluation of the stool was negative. Bowel wall thickening was deemed to be an infectious versus reactive process and the patient was discharged on antibiotics and proton pump inhibitors.

**Figure 2:**
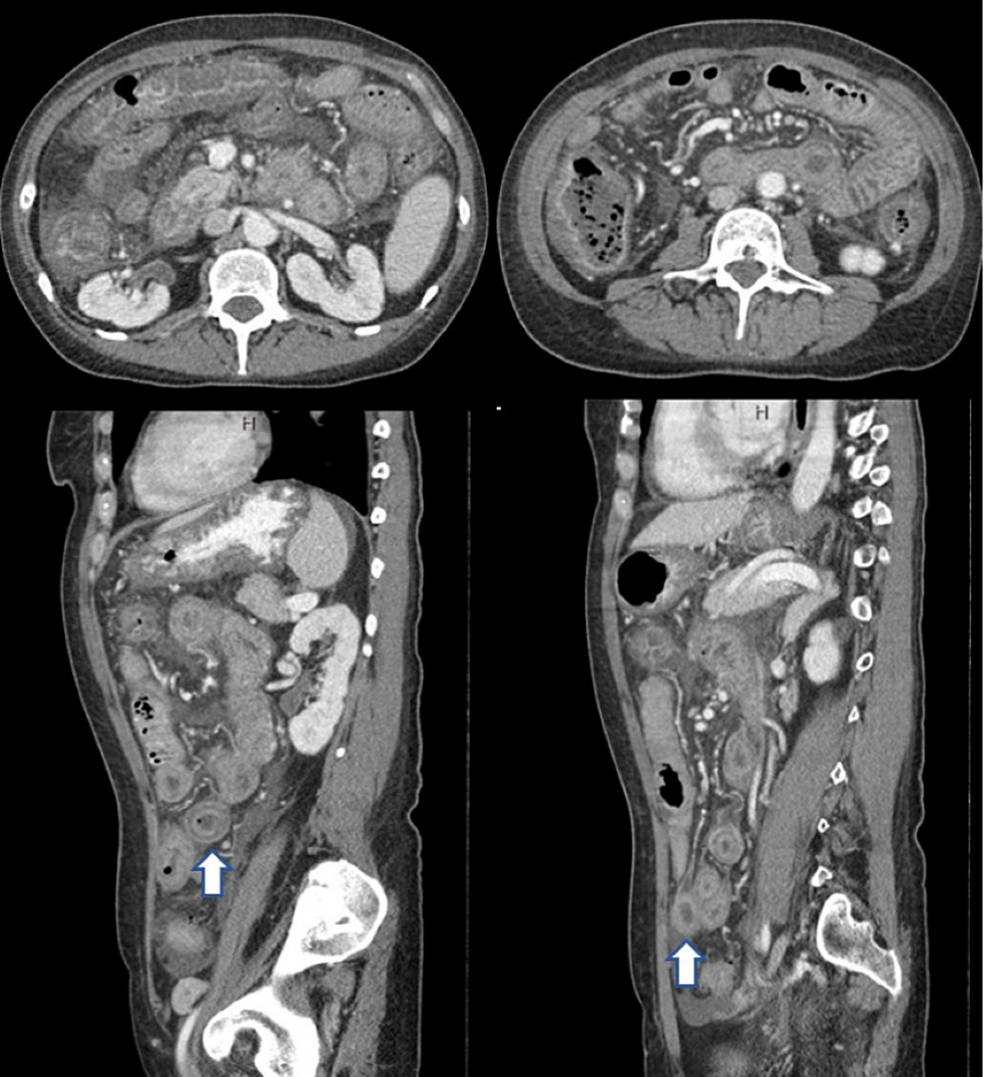
Contrast-enhanced abdominal CT scan - case 2 Diffuse thickening of the small intestine and colon wall with target sign seen (arrows) CT: computed tomography

The patient later presented to our hospital with persistent symptoms. Her vital signs were normal; she had generalized abdominal pain without guarding or rebound tenderness, and no synovitis or rash was present. Initial evaluation revealed hyponatremia (sodium: 132 mEq/L), hypomagnesemia (serum magnesium: 1.6 mg/dl), hypoalbuminemia (albumin: 3.3 g/dl), thrombocytopenia (platelet count: 124/ul), lymphopenia (200 k/µL), and elevated CRP (0.67 mg/dl). Repeat infectious evaluation was negative. Celiac disease antibodies (TTG IgG/IgA, anti-gliadin) were negative as well. Further evaluation revealed ANA titer of >1:160, C3 of <50 mg/dl, C4 of <6 mg/dl, positive anti-Smith of >8, anti-dsDNA of 4 IU/ml, and SSA of >8. CT imaging re-demonstrated diffuse thickening and enhancement of the small bowel and new diffuse wall thickening and submucosal edema of the entire colon and rectum with small-volume ascites (Figure 2). Despite symptomatic treatment, the patient's status remained unchanged. Lupus enteritis was diagnosed and she was started on methylprednisolone 32 mg IV for five days with significant improvement. She was later transitioned to hydroxychloroquine 200 mg daily, azathioprine (50 mg daily, later increased to 100 mg), and prednisone 60 mg daily (tapered off over three months). The patient remained asymptomatic at the three-month clinic follow-up.

## Discussion

GI symptoms in SLE patients are common. They are usually attributable to medication side effects, infections, or other associated conditions (autoimmune hepatitis, primary biliary cirrhosis, inflammatory bowel disease, or celiac disease) [5]. Less commonly, they can be caused by SLE itself [1]. Both older [American College of Rheumatology (ACR), 1982, revised 1997] and new [European League Against Rheumatism (EULAR) and ACR, 2019] classification criteria for SLE do not include GI symptoms [6,7].

Lupus enteritis is defined by the British Isles Lupus Assessment Group (BILAG, 2004) as either vasculitis or inflammation of the small bowel wall that is supported by either imaging or biopsy findings [8]. The mechanism is believed to involve immune complex deposition and thrombosis of the intestinal vessels [9,10]. It is unusual for the GI tract to be involved in the absence of other symptoms of lupus since other organs are generally affected first. Therefore, patients with lupus enteritis usually have high disease activity scores on scales like SLE Disease Activity Index (SLEDAI) or BILAG [1,9]. According to Janssens et al., only 13% of patients with lupus enteritis were simultaneously diagnosed with SLE for the first time [9]. In the study conducted by Kwok et al., SLEDAI scores and the mean steroid dose prior to presentation were higher for patients diagnosed with lupus enteritis compared to SLE patients with abdominal pain secondary to other causes [4].

Most patients with this condition are female. Symptoms are nonspecific, including abdominal pain (97%), ascites (78%), nausea (49%), vomiting (42%), and diarrhea (32%) [9]. Jejunum and ileum are the most commonly affected segments (83% and 84%, respectively); however, the entire GI tract can be involved [9,11,12].

Maruyama et al. (2018) described two types of disease among Japanese patients: small intestine-dominant (small bowel with or without cecum/ascending colon) and large intestine-dominant (more than one segment of large bowel regardless of small bowel involvement). The latter resembles intestinal pseudo-obstruction and is associated more often with older age and extra-intestinal symptoms such as hydroureter, lupus cystitis, or bile duct disease. The first type was more frequently seen with biopsy-proven lupus nephritis and more typical features of SLE [13]. Lupus nephritis commonly co-exists with lupus enteritis and it is important to exclude it [1,9]. Lupus cystitis has also been associated with lupus enteritis; patients present with urinary symptoms and, sometimes, obstructive uropathy due to edema and fibrosis at the ureterovesical junction [3,13,14].

The most common laboratory findings of lupus enteritis include hematologic derangements (leukopenia, lymphopenia, and anemia), positive ANA (92%), anti-dsDNA (74%), low complement (88%), anti-RNP (28%), anti-SSA (26%), and anti-Sm (24%) [9]. CRP elevation is not characteristic of the disease [9]. Specific autoantibodies related to lupus enteritis have not been identified.

Abdominal CT scan is considered the first-line diagnostic modality [1,9]. There are three classic findings: bowel wall edema and enhancement (“target sign”), engorgement/increased number of mesenteric vessels (“comb sign”), and increased attenuation of mesenteric fat [15,16]. Ascites is also commonly present. These findings are nonspecific and can also be seen with other conditions, such as intestinal obstruction, pancreatitis, or inflammatory bowel disease [11]. Coordination with the radiology department is important as the radiologist might not be aware of the presence of SLE in clinical history, which can lead to delayed diagnosis. Abdominal ultrasound can be used when CT is not available [17,18]. Luis et al. described seven cases of lupus enteritis where abdominal ultrasound was performed; in all of them, bowel wall thickening, dilation of the intestinal segments, and mild ascites were present [14]. There have been reports of the use of MRI/magnetic resonance enterography (MRE) with good performance; however, it is costly and time-consuming [18,19]. Biopsies usually include only superficial layers and the findings are nonspecific; only some patients have findings of vasculitis or necrosis. Endoscopy studies help exclude other etiologies but are not considered useful for diagnosis. Nonspecific findings like mucosal edema, erosions, and ulcers have been reported to be associated with lupus enteritis [9].

First-line treatment includes systemic corticosteroids. Janssens et al. recommend methylprednisolone 250 mg-1 g IV per day followed by oral prednisone 0.5-1 mg/kg per day if tolerable or initial therapy with prednisone in case of mild symptoms [9]. Appropriate bowel rest, hydration, and electrolyte repletion are essential supplementary therapies. Most patients experience symptomatic improvement with corticosteroid therapy [9,11]. Cyclophosphamide, azathioprine, or mycophenolate mofetil are added for more severe and relapsing disease [9,10]. These agents, as well as hydroxychloroquine, can be used as long-term maintenance therapy. There have been cases of successful management of severe lupus enteritis unresponsive to other treatment methods with intravenous cyclophosphamide pulses according to the Euro-Lupus protocol [20]. There are also reports of successful treatment with rituximab in refractory cases [3,19].

The most serious complications are life-threatening and include bowel wall ischemia and perforation. Suspicion for perforation should be high and warrants close clinical and radiological monitoring since systemic corticosteroids can mask signs of peritonitis [2]. Regarding the risk of recurrence, Koo et al. have identified colon involvement and the presence of lupus cystitis as the main risk factors [3]. Bowel wall thickness exceeding 8-9 mm has also been associated with recurring disease [15].

## Conclusions

Lupus enteritis is a rare presentation of SLE. It is even rarer for it to be the only clinical feature of SLE flare. Symptoms are nonspecific and the CT scan of the abdomen is the diagnostic modality of choice. It is usually responsive to corticosteroids and immunosuppressants. Our cases highlight this unusual manifestation as the only symptom of active SLE. A high index of suspicion should be maintained when evaluating SLE patients presenting with GI symptoms to prevent diagnosis and treatment delays that could lead to serious complications such as bowel necrosis, perforation, and even death.
